# RIOK2 Inhibitor NSC139021 Exerts Anti-Tumor Effects on Glioblastoma via Inducing Skp2-Mediated Cell Cycle Arrest and Apoptosis

**DOI:** 10.3390/biomedicines9091244

**Published:** 2021-09-17

**Authors:** Min Yu, Xiaoyan Hu, Jingyu Yan, Ying Wang, Fei Lu, Junlei Chang

**Affiliations:** 1 Shenzhen Key Laboratory of Biomimetic Materials and Cellular Immunomodulation, Institute of Biomedicine and Biotechnology, Shenzhen Institute of Advanced Technology, Chinese Academy of Sciences, Shenzhen 518055, China; yuminwanan@163.com (M.Y.); huxiaoyan18@foxmail.com (X.H.); jingyuyu916@163.com (J.Y.); wangying@siat.ac.cn (Y.W.); 2 University of Chinese Academy of Sciences, Beijing 100864, China; 3 State Key Laboratory of Chemical Oncogenomics, Peking University Shenzhen Graduate School, Shenzhen 518055, China; 4 Key Laboratory of Chemical Genomics, Peking University Shenzhen Graduate School, Shenzhen 518055, China

**Keywords:** glioblastoma, NSC139021, cell cycle, Skp2 pathway, proliferation, apoptosis

## Abstract

Up to now, the chemotherapy approaches for glioblastoma were limited. 1-[2-Thiazolylazo]-2-naphthol (named as NSC139021) was shown to significantly inhibit the proliferation of prostate cancer cells by targeting the atypical protein kinase RIOK2. It is documented that RIOK2 overexpressed in glioblastoma. However, whether NSC139021 can inhibit the growth of glioblastoma cells and be a potential drug for glioblastoma treatment need to be clarified. In this study, we investigated the effects of NSC139021 on human U118MG, LN-18, and mouse GL261 glioblastoma cells and the mouse models of glioblastoma. We verified that NSC139021 effectively inhibited glioblastoma cells proliferation, but it is independent of RIOK2. Our data showed that NSC139021 induced cell cycle arrest at G0/G1 phase via the Skp2-p27/p21-Cyclin E/CDK2-pRb signaling pathway in G1/S checkpoint regulation. In addition, NSC139021 also increased the apoptosis of glioblastoma cells by activating the p53 signaling pathway and increasing the levels of Bax and cleaved caspase 3. Furthermore, intraperitoneal administration of 150 mg/kg NSC139021 significantly suppressed the growth of human and mouse glioblastoma in vivo. Our study suggests that NSC139021 may be a potential chemotherapy drug for the treatment of glioblastoma by targeting the Skp2-p27/p21-Cyclin E/CDK2-pRb signaling pathway.

## 1. Introduction

Glioblastoma (GBM) is one of the most common and aggressive primary malignant neoplasms of the central nervous system with a high mortality rate [1,2]. The current standard treatment includes surgical resection, followed by a combination of radiation and chemotherapy with temozolomide (TMZ) [3,4]. Despite extensive efforts to develop new treatment strategies, the median survival for patients with GBM is 15 to 20 months from the time of diagnosis, and only 3% to 5% of patients survive longer than 5 years [5]. TMZ as a DNA alkylating agent targeting proliferating cells only exhibited modest efficacy, which in combination with radiation can only increase the survival time by about 3 months compared to radiotherapy alone [6,7,8] and improve the 2-year survival rate from 10% to 26% [7]. Further, TMZ treatment demonstrates potent side effects, low response rate, and drug resistance, which significantly limit the clinical value of TMZ [9,10]. Therefore, new chemotherapy drugs for GBM are urgently needed.

Small molecule compounds are an important source of drugs against various human diseases, including cancers. 1-[2-Thiazolylazo]-2-naphthol (named as NSC139021) belongs to thiazolyl-diazo-naphthol compounds, which consist of (i) thiazoyl ring, (ii) diazo linker, and (iii) naphthol ring, has shown significant growth inhibition effects on the ERG-positive prostate cancer cells [11]. Previously, NSC139021 was reported to exert its anti-tumor effects by directly interacting with RIOK2 and inducing ribosomal stress in prostate cancer. RIOK2 is a member of the RIO (right open reading frame) family of atypical protein kinases that also includes RIOK1 and RIOK3 [12]. Recent studies have revealed that RIO kinases are involved in glioma cell proliferation, expansion, migration, and invasion [13,14,15]. To date, several studies have demonstrated that RIOK2 is overexpressed in GBM tumor cells relative to normal brain cells [13,14,15], and RIOK2 loss causes a reduction in Akt signaling and provokes p53-dependent apoptosis, cell cycle exit, and chemosensitivity through the RpL11-dependent ribosomal stress checkpoint [13], which in turn suggests that RIOK2 is engaged in tumorigenic activity in GBM, can be a potential target for anti-GBM. However, whether NSC139021 can inhibit the growth of glioblastoma cells and the mechanisms are still unclear.

Here, we investigated the anti-glioblastoma effects of RIOK2 inhibitor NSC139021 and found that it inhibited the viability and proliferation of human and mouse glioblastoma cells via a mechanism independent of RIOK2 and other RIO kinases. Interestingly, we found that NSC139021 arrested the cell cycle at the G0/G1 phase by targeting the Skp2 (S-phase kinase-associated protein 2, also named p45)-p27/p21-Cyclin E/CDK2-pRb pathway and eventually lead to cell apoptosis. Skp2 belongs to the F-box protein family that has the potential to act as one of anti-tumor therapeutic targets and prognostic biomarkers via the proteolytic pathway. Skp2 regulates cell cycle progression through combining with Skp1, Cullin-1, and Rbx1 to form the Skp1-Cullin-1-F-box (SCF) multiprotein complex then induces the ubiquitination and degradation of substrates, such as cell cycle checkpoint inhibitors (p27, p21, and p57) [16,17,18,19,20]. Our results suggest that NSC139021 acts as a new inhibitor of the Skp2 pathway and may be a potential chemotherapeutic drug for the treatment of GBM.

## 2. Materials and Methods

### 2.1. Materials

NSC139021(HY-112158) was purchased from MedChemExpress (Monmouth Junction, NJ, USA) and was solubilized in dimethylsulfoxide (Sigma-Aldrich, St. Louis, MO, USA) at 20 mM and 75 mg/mL stock solution. Antibodies were as follows: anti-Cyclin A (sc-751), anti-CyclinB1 (sc-245), anti-Cyclin E (sc-25303), anti-Skp2 (sc-7164), anti-p-Rb (Ser807) (sc-293117), and anti-p27 (sc-528) were purchased from Santa Cruz Biotechnologies (Dallas, DE, USA); anti-RIOK1 (17222-1-AP), anti-RIOK3 (13593-1-AP), anti-GAPDH (60004-1-Ig), anti-p21 (10355-1-AP), and anti-β-actin (66009-I-Ig) were purchased from Proteintech (Wuhan, China); anti-p53 (2524S), anti-caspase-3 (9662S) were purchased from Cell Signaling Technology (Boston, MA, USA); anti-Bcl-2 (A19693) and anti-Bax (A19684) were purchased from ABclonal (Wuhan, China); anti-RIOK2 (HPA005681) was from Sigma-Aldrich (St. Louis, MO, USA); homemade polyclonal antibody of CDK2 was the gift from Dr. Hui Zhang (Department of Chemistry and Biochemistry, University of Nevada, Las Vegas, NV 89154, USA). Cell Counting Kit-8 (CCK-8) (BA00208) was obtained from Bioss (Beijing, China), and Annexin-V-FITC/PI kit (40302ES60) was purchased from YEASEN (Shanghai, China).

### 2.2. Cell Lines and Culture Conditions

Human glioblastoma cell lines U118MG and LN-18 were obtained from American Type Culture Collection (ATCC). Cells were maintained in Dulbecco’s modified Eagle’s medium (DMEM) with 10% FBS and 100 U/mL penicillin and 100 μg/mL streptomycin at, 37 °C, 5% CO_2_ incubator. Mouse GL261 glioblastoma cell line (derived from a C57BJ/L6 genetic background) was from the National Cancer Institute Tumor Repository and grown in DMEM supplemented with 10% FBS and 100 U/mL penicillin and 100 μg/mL streptomycin in humidified chambers at 37 °C with 5% CO_2_. Cells were authenticated by Shanghai Biowing Biotechnology Co. Ltd., and mycoplasma was tested after cells were cultured for one week using GMyc-PCR Mycoplasma Test Kit (YEASEN, Shanghai, China). Cells used for experiments were maintained for one month.

### 2.3. Proliferation Assays

Proliferation assays were performed using the CCK-8 kit and crystal violet. Briefly, when cells were in exponential growth, 100 μL of U118MG, LN-18, and GL261 cells were respectively seeded in 96-well plates at a density of 2 × 10^4^ cells/mL. After incubating for 24 h, cells were treated with NSC139021 (5, 10, 15 μM) and equivalent amounts of DMSO for 24, 48, or 72 h. A concentration of 10 μL CCK-8 was added to each well and cultured for 2 h in the dark. The absorbance of optical density was measured with a microplate reader (Multiskan GO, Thermo Fisher Scientific, Waltham, MA, USA). The experiments were performed in triplicate. U118MG, LN-18, and GL261 cells were plated onto the 12-well plates at 750 cells per well. After 24 h, cells were treated with NSC139021 for 48 h, and the final concentrations were 0, 5, 10, and 15 μM. Then, each well was washed by PBS, and the medium was changed to normal medium without NSC139021. After 10–14 days, each well was washed with PBS three times at room temperature, then fixed by 4% PFA for 10 min and finally stained with 0.3% crystal violet for 20 min at room temperature. The experiments were performed in triplicate.

### 2.4. Transfection and siRNAs

For siRNA-mediated gene silencing, cells were transfected with 50 nM siRNAs using LipofectamineTM RNAimax Transfection Reagent (#13778075, Thermo Fisher Scientific, Waltham, MA, USA) according to the product manual. To rule out potential off-target effects of siRNAs, three pairs of siRNAs for RIOK2 and RIO kinases were designed and synthesized by GenePharma Company (Suzhou, China). The sequences for siRNAs were listed in Supplemental Table S1.

### 2.5. Flow Cytometry Analysis of Cell Cycle and Apoptosis

Cell cycle was detected using PI staining as previous description [21]. U118MG and LN-18 cells were starved without serum for 24 h, then cultured in complete medium and treated with DMSO and NSC139021 (5, 10, 15 μM). After 24 h, cells were harvested by trypsinization and fixed with ice-cold 70% ethanol for 4 h at 4 °C. After being rinsed with PBS, cells were incubated in staining buffer (25 μg/mL propidium iodide (PI), 1% Triton X-100 and 50 μg/mL RNAase) for 45 min at 37 °C, and analyzed by flow cytometry (CytoFLEX S, Beckman Coulter, Brea, CA, USA). DNA contents were evaluated with FlowJo7.6.5. Each experiment was performed in triplicate.

Apoptosis was detected using AnnexinV-fluoroisothiocyanate (FITC)/PI staining. U118MG and LN-18 cells were treated with NSC139021 for 72 h. Cells were digested with 0.05% trypsin and centrifuged for 5 min. The collected cells were washed with cold PBS twice, and the precipitated cells were resuspended in 100 μL binding buffer costained with 5 μL Annexin-V-FITC and 10 μL PI, then incubated for 15–30 min in the dark. Stained cells were analyzed through flow cytometry (CytoFLEX S, Beckman Coulter, Brea, CA, USA).

### 2.6. Total RNA Isolation and Real-Time PCR

Total RNA was extracted using the Direct-zol RNA MiniPrep kit (R2052, Zymo Research). RNA was reverse transcribed using HiScript Reverse Transcription Supermix for RT-qPCR according to the manufacturer′s instructions (R323-01, Vazyme, Nanjing, China). RT-qPCR was carried out on a Real-Time PCR System using the Power SYBR Green method (Applied Biosystems). β-actin was taken for normalization. Gene-specific primer sequences were as followed:

β-actin: 5′-GCAAAGACCTGTACGCCAAC-3′ (forward)
and 5′-GATCTTCATTGTGCTGGGTGC-3′ (reverse);
Skp2: 5′-AGACTGGATGAGCTGAACCTCTCC-3′ (forward)
and 5′-GGTGATGGTCTCTGACACATGCG-3′ (reverse);
p27: 5′-ACTGAGGCGGAGACGAAGGTG-3′ (forward)
and 5′-CGCTGTTTGTCTTGGAGGAGGATC-3′ (reverse);
RIOK2: 5′-TCCAGGGCTATCGGTTGACAAATG-3′ (forward)
and 5′-TTGCCAACACCCATCTGGTTTCC-3′ (reverse).

### 2.7. Western Blot Analysis

The protein samples were denatured for 10–15 min at 100 °C. A total of 20~70 µg protein from each sample was separated on an SDS-PAGE gel and transferred to a nitrocellulose filter membrane. Membranes were blocked for 1 h at room temperature in 5% non-fat dry milk and then incubated overnight at 4 °C with primary antibodies diluted in TBS with 0.1% Tween 20 (TBST). Membranes were washed in TBST and then incubated with secondary antibodies (HRP-anti-Rabbit IgG, SA00001-1, Proteintech; HRP-anti-Mouse IgG, SA00001-2, Proteintech) for 1 h at room temperature. Subsequently, the membranes were exposed to enhanced chemiluminescence substrate detection solution (WBKLS0500, MillPore) and then detected by instrument (Gel view, GV6000).

### 2.8. Mouse Tumor Model and Treatment

Procedures involving mice were approved by the Institutional Animal Care and Use Committee at the Shenzhen Institute of Advanced Technology, Chinese Academy of Sciences, in compliance with the Guide for the Care and Use of Laboratory Animals (SIAT-IRB-170304-YYS-CJL-A0300, 8 February 2018). Female nude mice (5–6 weeks) and C57BJ/L6 mouse (10–12 weeks) were obtained from Beijing Vital River Laboratory Animal Technology Co., Ltd., and all mice were housed in barrier facilities with a 12 h light/dark cycle. Human U118MG glioblastoma cells (5 × 10^6^ cells) in PBS were subcutaneously injected into the right flanks of the mice. After 2 weeks, the mice were randomly divided into three groups (*n* = 10) and treated three times per week for 21 days with NSC139029 (100 and 150 mg/kg, body weight, about 50–75 μL/mouse, i.p.) or vehicle control (1:1 (*v*/*v*), DMSO/PEG300) [11]. An orthotopic glioblastoma model was performed as we previously reported [22]. GL261 cells were trypsinized and resuspended at 10,000 cells per μL of PBS. A total of 30000 cells were implanted into the striatum, 1.8 mm to the right of the bregma, at a depth of 2.8 mm using a stereotactic injection frame over the course of 5 min. The needle was slowly withdrawn at a pace of 0.5 mm every 30 s, and the burr hole was sealed with bone wax before the closure of the skin with a 4–0 silk suture. After one week, mice were randomly separated into experimental group and control group, 5~6 mice in each group. In the treatment group, mice were intraperitoneally injected with 150 mg/kg of NSC139021, while the control group was injected with vehicle only. Mice were euthanized at 17 days of treatment [11].

### 2.9. Quantification and Statistical Analysis

Experimental data were analyzed using the unpaired, two-tailed Student’s t-test. Results represented as *p* < 0.05 were considered statistically significant. The densitometry quantitation of the bands was performed using Image J software. All figures and graphs were elaborated with Adobe Photoshop CS5 and Graph Pad Prism 8.0.

## 3. Results

### 3.1. NSC139021 Inhibits Glioblastoma Cell Proliferation through a RIOK2-Independent Mechanism

**Figure 1:**
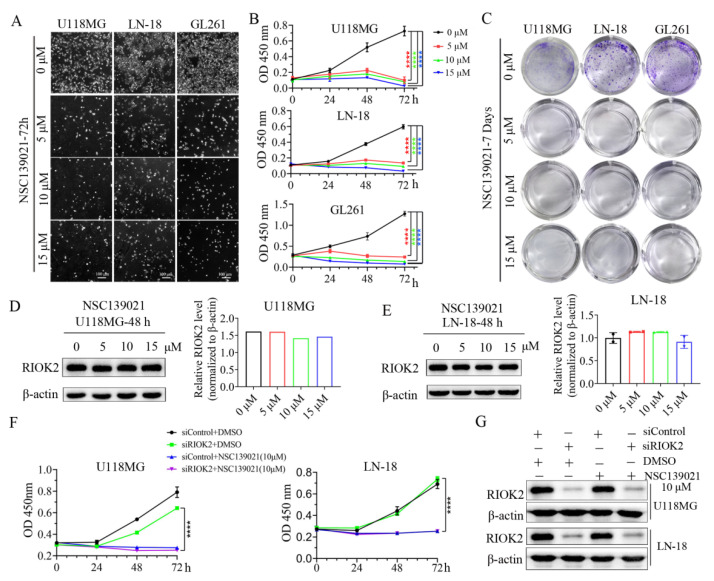
NSC139021 inhibited the proliferation of glioblastoma cells independently on RIOK2. (**A**) Cell proliferation was inhibited after NSC139021 (5, 10, 15 μM) treatment for 72 h in U118MG, LN-18, and GL261 cells. (**B**) CCK-8 assays showed that cell viabilities were decreased after NSC139021 (5, 10, 15 μM) treatment for 48 h or 72 h in U118MG, LN-18, and GL261 cells. (**C**) Colony formation assays also indicated that colony numbers were decreased after NSC139021 (5, 10, 15 μM) treatment for 10–14 days in U118MG, LN-18, and GL261 cells. (**D**,**E**) RIOK2 protein level in U118MG and LN-18 cells after treatment with NSC139021 and was quantified and normalized to β-actin and plotted on the right panel. (**F**) CCK-8 assays demonstrated that neither cell viabilities nor the inhibitory effects of NSC139021 (10 μM) on proliferation were affected by siRIOK2 in U118MG and LN-18 cells. (**G**) Western blot analysis showed that RIOK2 was reduced by siRIOK2 interfering for 72 h but was not affected by NSC139021 (10 μM) treatment for 48 h. **** *p* < 0.0001 when compared to the DMSO group.

To elucidate the role of NSC139021 on glioblastoma proliferation, we selected concentrations of 5, 10, and 15 μM NSC139021 to investigate its anticancer effects and mechanism. After treatment for 72 h, cell number was significantly reduced in U118MG, LN-18, and GL261 cells (Figure 1A). Treatment with 5, 10, and 15 μM NSC139021 for 24, 48, and 72 h resulted in a dose- and time-dependent inhibition of proliferation of U118MG, LN-18, and GL261 cells by using CCK-8 kit (Figure 1B). Plate colony formation assays showed that the number of colony formation was decreased after 2 days’ treatment in U118MG, LN-18, and GL261 cells (Figure 1C). These results indicated that glioblastoma cell proliferation was inhibited by NSC139021 treatment in a dose- and time-dependent manner. To test the inhibitory effects of NSC139021 on RIOK2 protein level in glioblastoma cells, we treated cells with NSC139021 for 48 h. Western blot results showed that NSC139021 had no effect on the protein level of RIOK2 (Figure 1D,E). To further validate whether RIOK2 mediated the inhibitory effects of NSC139021 on glioblastoma cell proliferation, we silenced RIOK2 by using gene-specific siRNAs in U118MG and LN-18 cells. To rule out the off-target effects of siRNAs, we designed three pairs of siRNAs targeting RIOK2. Verified that each pair of siRNAs has similar efficacy and phenotype on glioblastoma cells (Supplemental Figure S1A,B), we used the siRIOK2-3 in subsequent experiments. Interfering expression of RIOK2 with 24 h (Supplemental Figure S2B), followed by treatment with 10 μM NSC139021 for 24, 48, and 72 h, resulted in knockdown of RIOK2 did not affect the inhibitory effects of NSC139021 in U118MG and LN-18 cells (Figure 1F,G and Supplemental Figure S2A). Thus, NSC139021 suppressed viability and proliferation of human glioblastoma cells was independent of RIOK2.

### 3.2. NSC139021 Induces Cell Cycle Arrest in G0/G1 Phase

**Figure 2:**
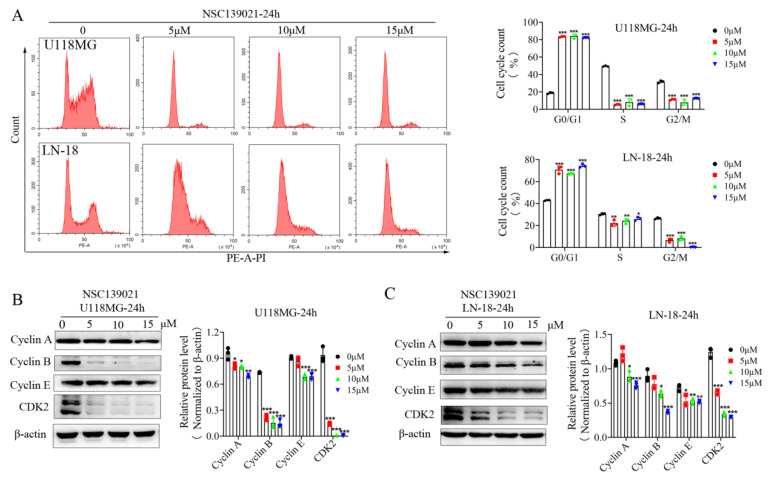
NSC139021-induced G0/G1-phase cell cycle arrest in glioblastoma cells. (**A**) Flow cytometric analysis with PI staining showed that the percentage of the G1 phase significantly increased, and the percentage of the S phase, as well as the G2/M phase, decreased after NSC139021 (5, 10, 15 μM) treatment in U118MG and LN-18 cells. Quantification of cell populations was plotted on the right panel. (**B**) Western blot analysis showed that NSC139021 (5, 10, 15 μM) treatment inhibited the expression levels of cell cycle-related proteins (Cyclin A, B, E, and CDK2) in U118MG cells. (**C**) Western blot analysis also showed that NSC139021 (5, 10, 15 μM) treatment inhibited the expression levels of cell cycle-related proteins (Cyclin A, B, E, and CDK2) in LN-18 cells. β-actin was used as an internal control. The protein levels were quantified and normalized to β-actin and plotted on the right panel. The data were represented as the mean ± SD of three independent experiments. * *p* < 0.05, ** *p* < 0.01, *** *p* < 0.001 when compared to the DMSO group.

Given that NSC139021 inhibits the cell viability of glioblastoma cells, identifying the underlying pathways involved is critical. Next, to investigate the mechanism of NSC139021 on inhibiting glioma cell growth, DNA-based cell cycle analysis with PI staining was performed on U118MG and LN-18 cells by flow cytometry after NSC139021 treatment. U118MG and LN-18 cells were starved without serum for 24 h, then cultured in complete medium and treated with DMSO and various concentrations of NSC139021. After treatment for 24 h, the cell cycle was almost blocked in G0/G1 phase in U118MG and LN-18 cells (Figure 2A). More clearly, the percentage of G0/G1 phase was accumulated to ~80%, ~70%, while that in control cells was ~20%, ~40% in U118MG and LN-18 cells, respectively (Figure 2A). Subsequently, we tested the effect of NSC139021 treatment on the proteins related to the cell cycle and G1-S-phase transition. Western blot analysis demonstrated that the dose-dependently downregulated the protein levels of Cyclin A and Cyclin B1 (Figure 2B,C), which was consistent with the cell cycle analysis and suggested that G2/M phase cell population was decreased after treatment for 24 h in U118MG and LN-18 cells. Cyclin E/CDK2 complex regulates the G1-S transition in the cell cycle [23]. Western blot results showed that CDK2 and Cyclin E were downregulated after treatment for 24 h in U118MG and LN-18 cells (Figure 2C). The above results indicated that NSC139021 inhibits G1-S-phase transition in U118MG and LN-18 cells. Taken together, our results indicated that NSC139021 arrested the cell cycle at G0/G1 phase, thereby inhibiting cell proliferation.

### 3.3. NSC139021 Arrests Cell Cycle by Regulating the Skp2-p27/p21-CDK2-Rb Signaling Pathway

**Figure 3:**
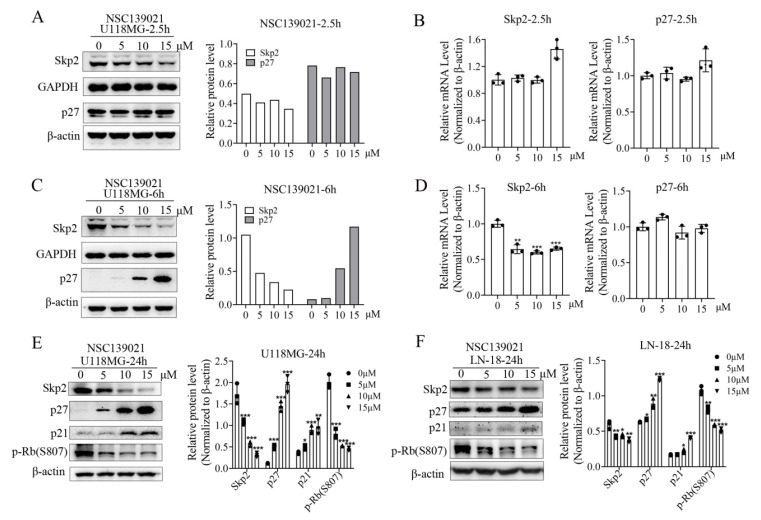
NSC139021 suppressed G1/S transition and cell cycle progression in glioblastoma cells via inhibiting the Skp2-p27/p21 axis. (**A**) Western blot analysis showed that NSC139021 (5, 10, 15 μM) treatment for 2.5 h downregulated the protein expression level of Skp2 but did not affect CDK inhibitor p27. The protein level was quantified and normalized to GAPDH or β-actin and plotted on the right panel. (**B**) Real-time PCR analysis showed that NSC139021 (5, 10, 15 μM) treatment for 2.5 h did not affect the mRNA expression level of Skp2 and p27. (**C**) Western blot analysis showed that NSC139021 (5, 10, 15 μM) treatment for 6 h also downregulated the protein expression level of Skp2 and upregulated the protein expression level of p27. The protein level was quantified and normalized to GAPDH or β-actin and plotted on the right panel (**D**). Real-time PCR analysis showed that NSC139021 (5, 10, 15 μM) treatment for 6 h downregulated the mRNA expression level of Skp2 but did not affect p27. (**E**,**F**) Western blot analysis showed that NSC139021 (5, 10, 15 μM) treatment for 24 h also downregulated the protein expression level of Skp2 and p-Rb (S807), upregulated the protein expression level of p27 and p21 in U118MG and LN-18 cells. β-actin was used as an internal control. The protein levels were quantified and normalized to β-actin and plotted on the right panel. The data were represented as the mean ± SD of three independent experiments.* *p* < 0.05, ** *p* < 0.01, *** *p* < 0.001 when compared to the DMSO group.

In addition to the cyclins and the activity of cyclin-dependent kinases (CDKs), protein degradation, which occurs through ubiquitin ligases such as TrCP and SCF complexes (SKP1-CUL1-Skp2 protein) [24], also plays an important role in the regulation of the cell cycle. To determine the mechanism of NSC139021 on blocking cell cycle in G0/G1 phase, glioblastoma cells were treated with NSC139021 after being starved without serum for 24 h. After treatment for 2.5 h, western blot and qPCR results showed that NSC139021 has no/slightly effect on the protein and mRNA expression level of Skp2 and its direct substrate p27 in U118MG cells (Figure 3A,B). While after treatment for 6 h, the protein and mRNA expression levels of Skp2 were decreased, followed by elevated p27 in a dose-dependent manner in U118MG cells (Figure 3C,D). After treatment for 24 h, the expression level of Skp2 was significantly downregulated, followed by elevated p27 and p21 in U118MG and LN-18 cells (Figure 3E,F). In addition, the phosphorylation level of G1/S checkpoint Rb also decreased by NSC139021 in U118MG and LN-18 cells (Figure 3E,F). These results indicated that the effect of NSC139021 blocked cell cycle in G0/G1 phase was time- and dose-dependent manner through regulating the Skp2-p27/p21-CDK2-Rb axis of G1/S checkpoint in glioblastoma cells.

### 3.4. NSC139021 Activates p53 Signaling Pathway and Triggers Apoptosis of Glioblastoma Cells

**Figure 4:**
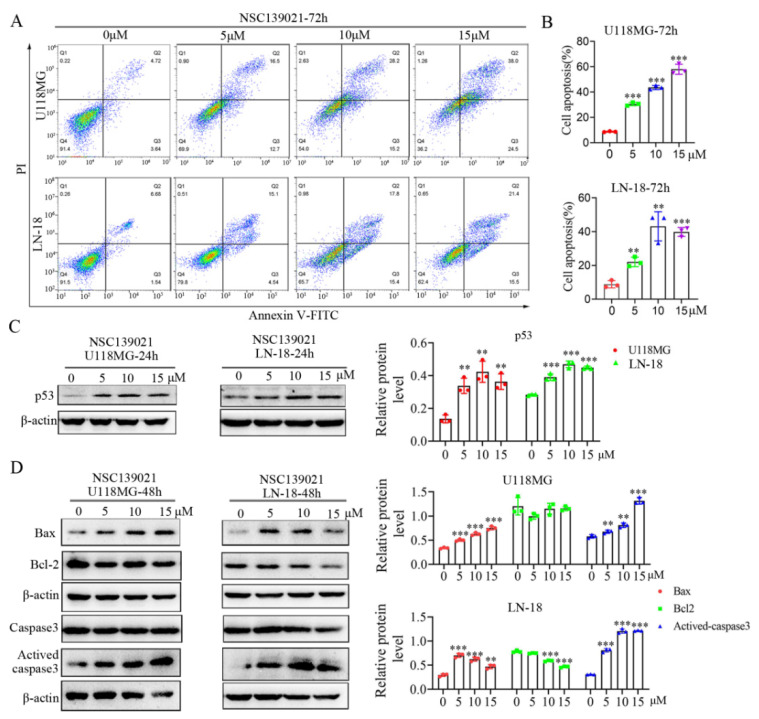
NSC139021-induced apoptosis of glioblastoma cells via activating the p53 signaling pathway. (**A**) Flow cytometric analysis with Annexin V and PI staining indicated that NSC139021 (5, 10, 15 μM) treatment for 72 h increased the percentage of apoptosis rate in U118MG and LN-18 cells. (**B**) Quantification of cell apoptosis was shown. Cells in Q2 and Q3 were counted. (**C**) Western blot analysis showed that NSC139021 (5, 10, 15 μM) treatment for 24 h upregulated the protein expression level of the apoptosis-associated protein p53 in U118MG and LN-18 cells, β-actin was used as an internal control. The protein level was quantified and normalized to β-actin and plotted on the right panel. (**D**) Western blot analysis showed that NSC139021 (5, 10, 15 μM) treatment for 48 h increased the expression levels of activated caspase and Bax in U118MG and LN-18 cells, slightly downregulated the expression level of Bcl-2 in LN-18 cells but not in U118MG cells, β-actin was used as an internal control. The protein levels were quantified and normalized to β-actin and plotted on the right panel. The data were represented as the mean ± SD of three independent experiments. ** *p* < 0.01, *** *p* < 0.001 when compared to the DMSO group.

Given that NSC139021 arrests the cell cycle of glioblastoma cells, we further asked whether NSC139021 can induce cell death of glioblastoma cells. Using flow cytometry analysis under Annexin V-FITC/ PI staining, we tested the effect of NSC139021 on apoptosis induction in U118MG and LN-18 cells. NSC139021 significantly induced apoptosis of these cells in a dose-dependent manner (Figure 4A,B). The apoptosis-associated p53 signaling was upregulated in U118MG and LN-18 cells with dose-dependent (Figure 4C). To investigate whether the induction of apoptosis was associated with caspase activation, we detected the expression of activated caspase3, Bcl-2, and Bax in U118MG and LN-18 cells upon NSC139021 treatment using western blotting. We found that NSC139021 dose-dependently induced Caspase3 activation and Bax upregulation (Figure 4D). These results indicated that NSC139021 triggered apoptosis in glioblastoma cells, and this was associated with p53-caspase3 activation.

### 3.5. NSC139021 Suppresses the Proliferation of Glioblastoma In Vivo

**Figure 5:**
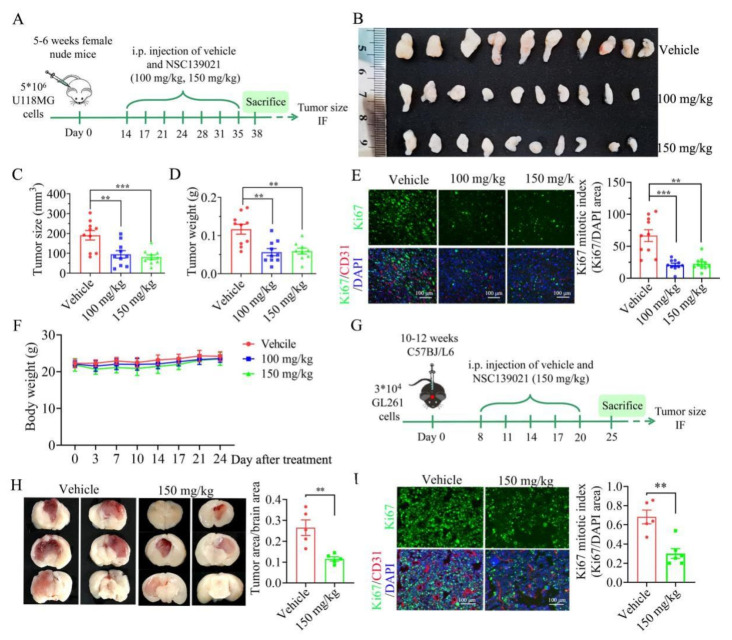
NSC139021 treatment inhibited the growth of glioblastoma in vivo. (**A**) The schematic representation of glioblastoma model with subcutaneous injection of U118MG cells. (**B**) Images of xenograft tumors in vehicle and NSC139021 (100 and 150 mg/kg) groups (*n* = 10 per group). (**C**) Tumor size at the end of the experiment (*n* = 10 per group). (**D**) Tumor weights at the end of the experiment (*n* = 10 per group). (**E**) Ki67/CD31/DAPI immunofluorescence (IF) staining of U118MG tumor (left panel). Quantification of Ki67-positive cells by calculating the ratio of the Ki67 IF staining-positive area to the DAPI area (right panel). (**F**) Changes in body weight during the NSC139021 administration period. (**G**) The schematic representation of glioblastoma model with orthotopic transplantation of GL261 cells. (**H**) Images of GL261 tumors in vehicle and NSC139021 (150 mg/kg) groups. Image J analysis indicated the ratio of tumor size with brain size was decreased in the NSC139021 group (*n* = 5 per group). (**I**) Ki67/CD31/DAPI co-IF staining of GL261 tumors (left panel). Quantification of Ki67-positive cells by calculating the ratio of the Ki67 IF staining-positive area to the DAPI area (right panel). (*n* = 5~6 per group). The data were represented as the mean ± SEM, ** *p* < 0.01, *** *p* < 0.001, when compared to the vehicle group.

To test the anti-tumor activity of NSC139021 against glioblastoma cells in vivo, we established a xenograft model by inoculating nude mice with U118MG cells. As a previous study reported that NSC139021 at a dose of 100 or 150 mg/kg did not cause obvious systemic toxicity in vivo [11], we treated nude mice with 100 or 150 mg/kg NSC139021 three times per week for 21 days (Figure 5A). Intraperitoneal administration of NSC139021 significantly decreased the volume and weight of subcutaneous U118MG xenograft tumors mainly by inhibiting the proliferation of glioblastoma cells in nude mice compared with vehicle controls (Figure 5B–E). After administration of NSC139021, no obvious weight loss or abnormal behavior was detected in nude mice (Figure 5F), which was consistent with a previous study [11]. To further investigate the anti-tumor activity of NSC139021 in orthotopic glioblastoma cells, we established a mouse GL261 orthotopic model (Figure 5G). Intraperitoneal injection of NSC139021 significantly decreased the size of the GL261 tumor and suppressed the proliferation of glioblastoma cells in C57BJ/L6 mice compared with vehicle control (Figure 5H,I). These results showed that NSC139021 has anti-tumor effects in subcutaneous and orthotopic glioblastoma models. Taken together, our results indicated that NSC139021 has anti-tumor effects in vivo.

## 4. Discussion

Small molecule compounds are an important source of drugs against various human diseases, including cancers, as a previous study reported that small molecule compound NSC139021 and its analogous compounds exert the anti-tumor therapeutic effects by directly interacting with RIOK2 and induced ribosomal stress signature in prostate cancer [11]. In this study, we investigated the anti-tumor activity of NSC139021 in glioblastoma cells and explored the underlying molecular mechanisms using the molecular characterization of signaling pathway regulation at both mRNA and protein levels. Our results showed that NSC139021 inhibited cell proliferation and colony formation independently on RIOK2 and other RIO kinases (RIOK1 and RIOK3) (Figure 1, Supplemental Figures S1 and S2). Our findings suggested that NSC139021 can arrest the cell cycle at G0/G1 phase and decrease the S phase and G2M phase cell population (Figure 2). The mechanism involved the Skp2-p27/p21-Cyclin E/CDK2-pRb signaling pathway in G1/S checkpoint regulation (Figure 2 and Figure 3, and Supplemental Figure S3). NSC139021 also increased the apoptotic rate by activating the p53 signaling pathway and increasing the expression of cleaved caspase 3 and Bax (Figure 4). Moreover, we found that intraperitoneal administration of 100 or 150 mg/kg NSC139021 suppressed the proliferation of mouse glioblastoma in vivo (Figure 5).

In eukaryotes, the cell cycle includes four phases (G0/G1, S, G2, and M), which are controlled by a complex series of signaling pathways, checkpoints, kinases, and other proteins. The dysregulated cell cycle of cancer cells resulted in uncontrolled cell proliferation and growth. At the G1/S checkpoint, the Cyclin E/CDK2 complex is crucial in regulating the G1/S-phase transition. Previous studies showed that the cell cycle checkpoint inhibitors p27/p21 suppressed the activity of Cyclin E/CDK2 and blocked the cell cycle at the G1 phase [24]. Skp2 as the E3 ligase, which regulates cell cycle progression through combining with Skp1, Cullin-1, and Rbx1 to form the Skp1-Cullin-1-F-box (SCF) multiprotein complex then induces the ubiquitination and degradation of substrates, such as p27, p21, p57 and p130 [16,17,18,25]. Earlier studies indicated that p27 degradation followed by phosphorylation on Thr-187 through Cyclin E/CDK2 complex can regulate the G1-S progression to take the oncogenic effect [26]. Skp2 is overexpressed in multiple types of tumors, including hepatocellular carcinoma, prostate cancer, and glioblastoma, and is related to cancer proliferation, invasion, and metastasis [27,28,29]. Furthermore, both upregulated Skp2 and downregulated p27 were identified to be correlated with poor prognosis in gastric carcinoma and neuroblastoma patients [30,31]. The Skp2-SCF complex inactivation with Cullin1 inhibitor MLN4924 suppressed tumorigenesis in p53/Pten-null PC3 prostate cancer cells [32]. Thus, the Skp2-p27 pathway is a critical target for cancer therapy. Our results indicated that the effect that NSC139021 arrested cell cycle in G0/G1 phase was time- and dose-dependent manner through regulating the Skp2-p27/p21-CyclinE/CDK2-pRb axis of G1/S checkpoint in glioblastoma cells and suggested that NSC139021 acted as a new potential inhibitor of Skp2 pathway and might be a chemotherapeutic drug in the treatment of GBM.

Previous studies showed that NSC139021 binds to RIOK2 and inhibits levels of ERG and RIOK2 protein in the context of ERG-positive cancer cells [11]. Due to the natural expression of RIOK2 and ERG in brain endothelial cells (data not shown), NSC139021 may affect the tumor vessels. Thus, it is important to note that a major conceptual challenge in systemic administration of NSC139021 has been related to blood vessel-associated toxicity [11].

**Figure 6:**
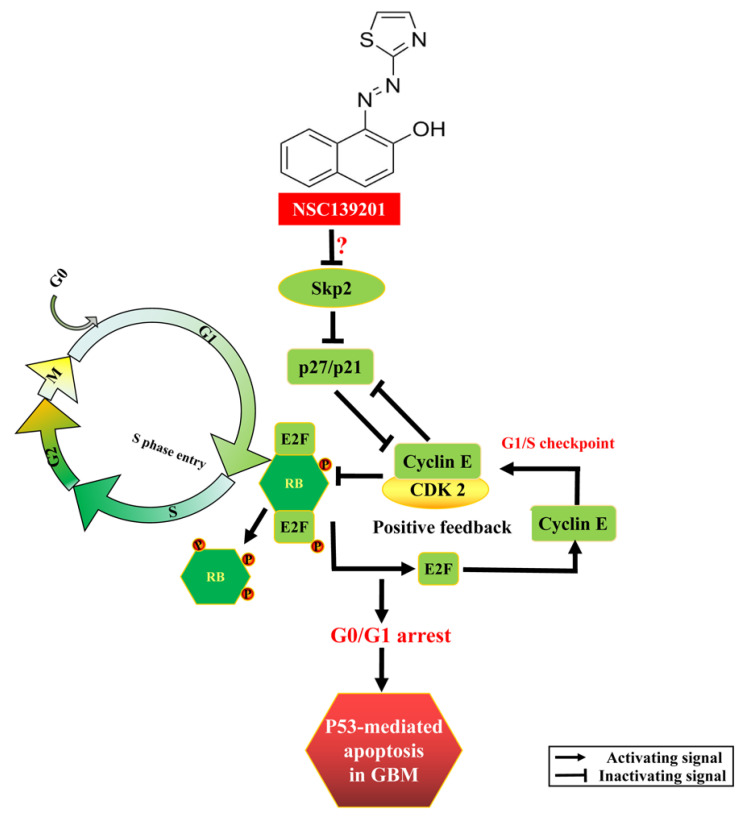
A schematic of the working mechanism of NSC139021 in glioblastoma. NSC139021 induces cell cycle arrest via the Skp2-p27/p21-Cyclin E/CDK2-pRb axis and triggers apoptosis via activating the p53 signaling. NSC139021 decreases the protein level of Skp2, which leads to the accumulation of p27 and p21. Subsequently, p27/p21 enhances the inhibition of CDK2, reduces the combination of CDK2 and Cyclin E, and decreases the dissociation of Rb and E2F. As the G1/S checkpoint, hyperphosphorylated Rb dissociates E2F that downregulates the expression of Cyclin E and forms positive feedback in the G1/S transition, and eventually activates the p53 pathway to induce cell apoptosis.

In conclusion, we found that NSC139021 exerted anti-tumor effects against glioblastoma by inducing p53-dependent apoptosis via cell cycle arresting at the G0/G1 phase (Figure 6). The mechanism involved regulation of the Skp2-p27/p21-Cyclin E/CDK2-pRb signaling pathway (Figure 6). This body of evidence supports the potential of NSC139021 as a chemotherapy drug in the treatment of glioblastoma.

## Data Availability

All data generated or analyzed during this study are included in the main body of this article.

## Supplementary Materials

The following are available online at https://www.mdpi.com/article/10.3390/biomedicines9091244/s1, Supplemental Figure S1: RIO kinases (RIOK1, RIOK2, and RIOK3) were downregulated by siRNAs in glioblastoma cells. Supplemental Figure S2: NSC139021 inhibited glioblastoma cell proliferation but was independent of RIO kinases. Supplemental Figure S3: Knockdown of RIOK2 had no effect on Skp2 protein level in glioblastoma cells. Supplemental Table S1: siRNAs sequences.
