# Supplementary material for: RIOK2 Inhibitor NSC139021 Exerts Anti-Tumor Effects on Glioblastoma via Inducing Skp2-Mediated Cell Cycle Arrest and Apoptosis
Source: Biomedicines. 2021 Sep 17;9(9):1244. doi: 10.3390/biomedicines9091244 (PMC8470931; doi:10.3390/biomedicines9091244)
Supplement: Supplementary file 1 [file biomedicines-09-01244-s001.zip › biomedicines-1333986-supplementary.pdf]

Supplementary Material

1 Supplemental Tables and Figures

1.1 Supplemental Tables

Supplemental Table S1. siRNAs sequences

Name	Sequences
siControl	UUCUCCGAACGUGUCACGUTT
siRIOK1-1	GGAAGCAACCCACAGGCAATT
siRIOK1-2	GCUAAUGUAUACCAUGCUATT
siRIOK1-3	GCUAAGAAGUCAUGUUCUUTT
siRIOK2-1	GGAUCUUGGAUAUGUUUAATT
siRIOK2-2	GGAUAUGAUUACCUAGCUUTT
siRIOK2-3	CCAGAUGGGUGUUGGCAAATT
siRIOK3-1	GCAAGUGUCCAUGGGCUAUTT
siRIOK3-2	GCAGGAUACUCGUGAUGAUTT
siRIOK3-3	GGCUGUAUUAGUACAGGAATT

1.2 Supplementary Figures

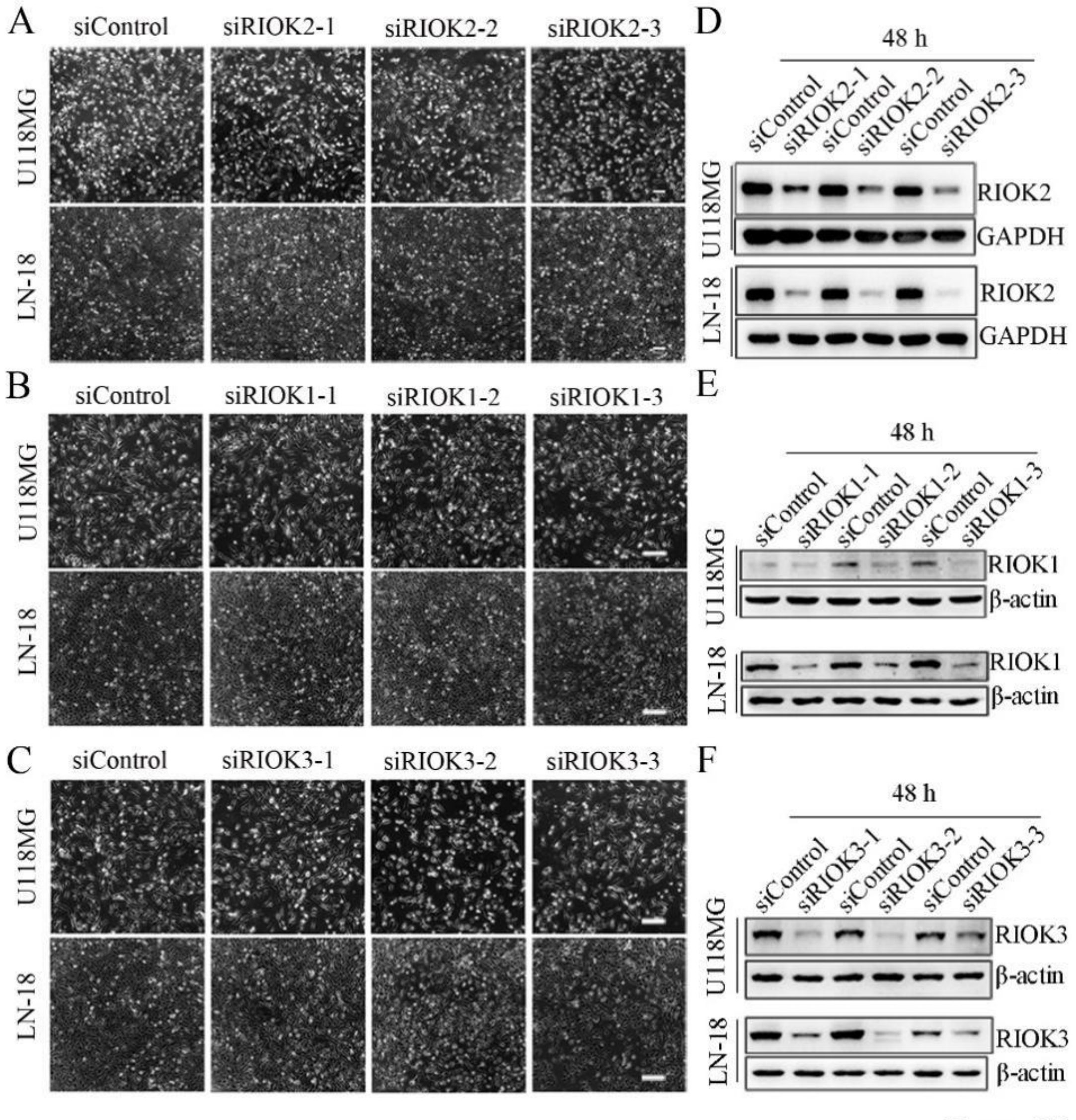

Supplemental Figure S1. RIO kinases (RIOK1, RIOK2, and RIOK3) were downregulated by siRNAs in glioblastoma cells. **(A-C)** Cell culture models showed that proliferation was not or slightly affected after siRNAs interfering for 48 h in U118MG and LN-18 cells. **(D-F)** Western blot results showed that the protein expression levels of RIO kinases were significantly decreased after siRNAs interfering for 48 h in U118MG and LN-18 cells, GAPDH and β-actin act as internal controls.

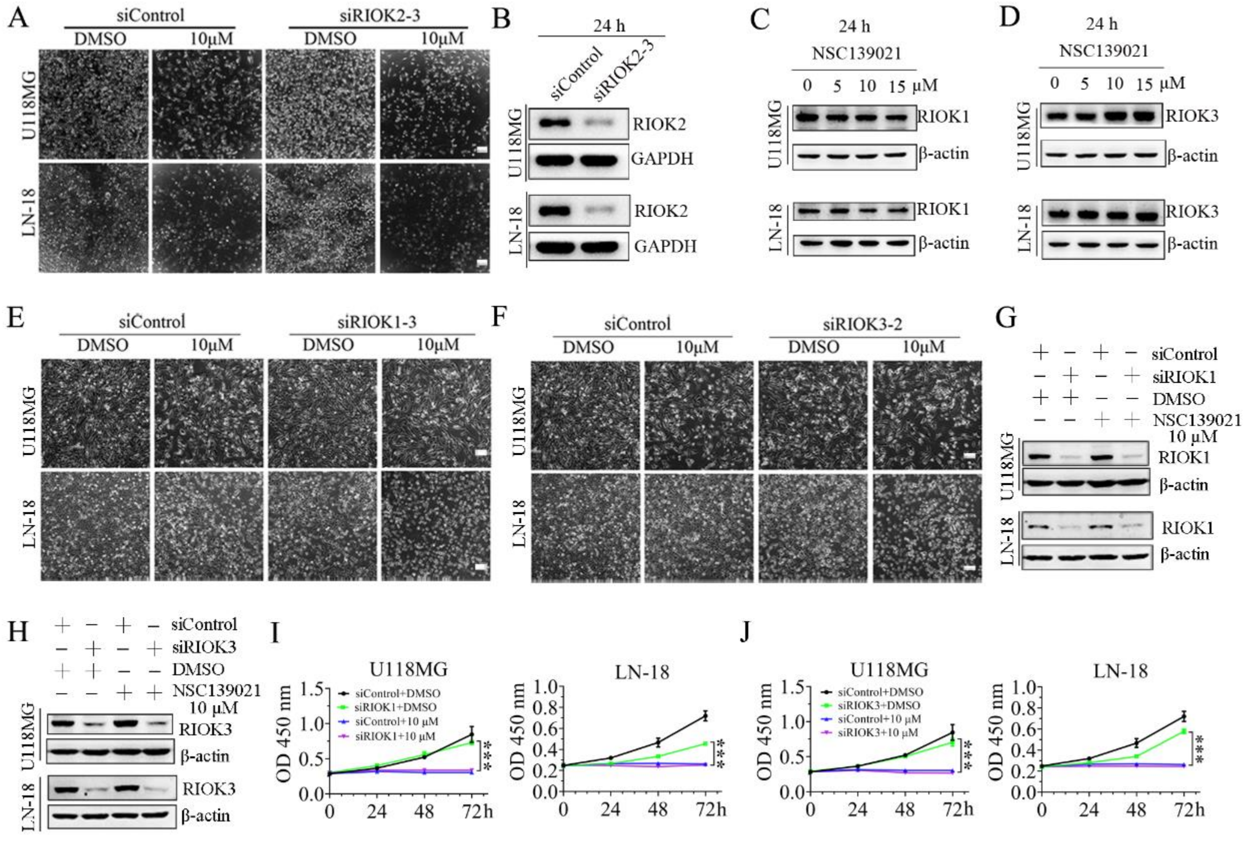

Figure S2

Supplemental Figure S2. NSC139021 inhibited glioblastoma cell proliferation but was independent on RIO kinases. **(A, E, F)** Cell culture models showed that NSC139021 (10μM) treatment for 72 h alone or with silenced RIO kinases took the similar inhibitory effects on proliferation in U118MG and LN-18 cells. **(B)** Western blot results showed that the protein expression level of RIOK2 was already significantly decreased after siRIOK2-3 interfering for 24 h in U118MG and LN-18 cells, GAPDH is an internal control. **(C, D)** Western blot analysis showed that RIOK1 or RIOK3 was not affected by NSC139021 (5,10, 15 μM) treatment for 24 h in U118MG and LN-18 cells, β-actin is an internal control. **(G, H)** Western blot analysis showed that RIOK1 or RIOK3 was silenced by siRNAs interfering for 72 h but was not affected by NSC139021 (10 μM) treatment for 48 h in U118MG and LN-18 cells. β-actin is an internal control. **(I, J)** CCK-8 assays demonstrated that neither cell viabilities nor the inhibitory effects of NSC139021 (10 μM) on proliferation were affected by siRIOK1 or siRIOK3 in U118MG and LN-18 cells. The data were represented as the mean ± SD of three independent experiments. ***P < 0.001 when compared to siRIOK1+DMSO or siRIOK3+DMSO group.

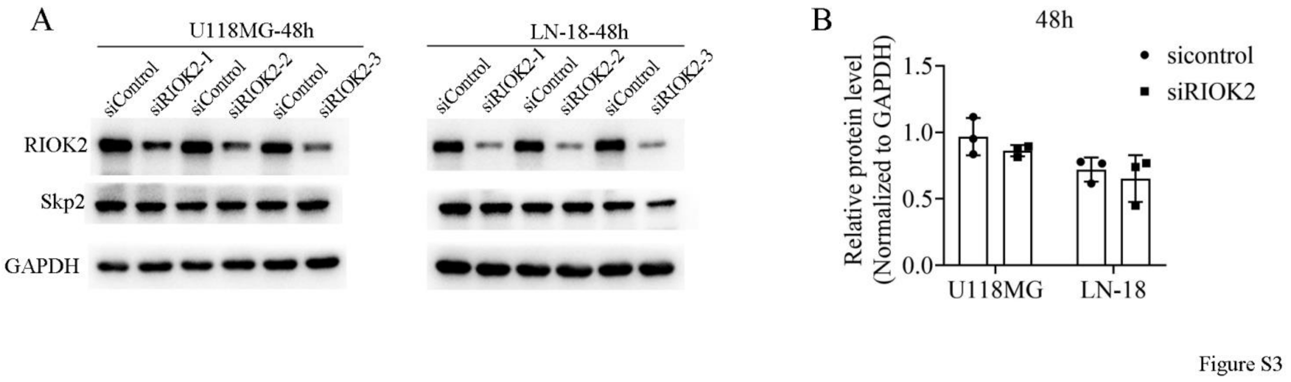

Supplemental Figure S3. Knockdown of RIOK2 had no effect on Skp2 protein level in glioblastoma cells. **(A)** Western blot analysis showed that Skp2 was not affected by silencing RIOK2 in U118MG and LN-18 cells. **(B)** The protein level of Skp2 was quantified and normalized to GAPDH.
